# Macular sub-layer thinning and association with pulmonary function tests in Amyotrophic Lateral Sclerosis

**DOI:** 10.1038/srep29187

**Published:** 2016-07-07

**Authors:** Joseph M. Simonett, Russell Huang, Nailah Siddique, Sina Farsiu, Teepu Siddique, Nicholas J. Volpe, Amani A. Fawzi

**Affiliations:** 1 Department of Ophthalmology, Northwestern University, Feinberg School of Medicine, Chicago, IL, USA; 2 Department of Neurology, Northwestern University, Feinberg School of Medicine, Chicago, IL, USA; 3 Departments of Biomedical Engineering and Ophthalmology, Duke University, Durham, NC, USA

## Abstract

Amyotrophic Lateral Sclerosis (ALS) is a complex neurodegenerative disorder that may have anterior visual pathway involvement. In this study, we compare the macular structure of patients with ALS to healthy controls, and examine correlations between macular sub-layer thickness measurements and pulmonary function tests and disease duration. ALS patients underwent optical coherence tomography (OCT) imaging to obtain macular cube scans of the right eye. Macular cube OCT data from age-matched healthy subjects were provided by the OCT reading center. Semi-automated retinal segmentation software was used to quantify macular sub-layers. Pulmonary function tests and time since symptom onset were collected retrospectively from the electronic medical records of ALS patients. Macular retinal nerve fiber layer was significantly thinner in ALS patients compared to healthy controls (*P* < 0.05). Total macular and other sub-layer thicknesses were not reduced in the ALS cohort. Macular retinal nerve fiber layer thickness positively correlated with forced vital capacity % predicted and forced expiratory volume in 1 second % predicted (*P* < 0.05). In conclusion, analysis of OCT measurements supports the involvement of the anterior visual pathway in ALS. Subtle structural thinning in the macular retinal nerve fiber layer correlates with pulmonary function tests.

Amyotrophic lateral sclerosis (ALS) is a neurodegenerative disorder of upper and lower motor neurons. Recently, studies have demonstrated cognitive impairment, whole-brain atrophy, and degeneration of non-motor systems in ALS^1^,^2^,^3^,^4^,^5^,^6^. Ocular motor dysfunction has been described in the disease, including gaze fixation instability, increased saccadic latency, smooth pursuit impairment, diminished Bell’s phenomenon, and eyelid apraxia^7^,^8^,^9^,^10^,^11^,^12^,^13^,^14^,^15^. However some studies have failed to confirm these findings and demonstrate preserved oculomotor function in ALS^16^,^17^. Additionally, some visual evoked potential (VEP) studies have demonstrated functional changes in sensory processing of the visual system in ALS cohorts, while other studies have reported normal VEPs^18^,^19^,^20^.

Although visual dysfunction is not a prominent feature of the ALS clinical presentation, decreased visual acuity and poor contrast sensitivity have been noted in a recent ALS cohort, prompting the hypothesis that subclinical structural defects in the anterior visual pathway may occur in ALS^14^.

Retinal and optic nerve structural defects have been demonstrated by optical coherence tomography (OCT) in various neurologic diseases including multiple sclerosis (MS), Parkinson disease (PD), and Alzheimer disease (AD)^21^,^22^,^23^,^24^. Several of these structural changes have been shown to correlate with disease duration and severity and have proven useful in differentiating phenotypically similar disorders, such as neuromyelitis optica and MS, as well as similar parkinsonian syndromes^25^,^26^,^27^,^28^,^29^.

Studies of OCT structural changes in ALS eyes have been limited, and to some extent heterogeneous. In 2014, Ringelstein *et al*. reported significantly decreased peripapillary retinal nerve fiber layer (RNFL), total macular, and inner nuclear layer (INL) thicknesses in a sporadic ALS population^30^. These findings differed from an earlier study that found no statistical differences in average peripapillary RNFL or macular sub-layer thicknesses between sporadic ALS patients and healthy controls^31^. Most recently, significant macular RNFL and INL thinning was found in a cohort of 71 ALS patients compared to 20 controls^32^. Interestingly, these authors also demonstrated a significant correlation between whole retinal thickness and fractional anisotrophy, a measure of axonal density, in the corticospinal tract. Correlations between OCT and pulmonary function testing, quantitative measures that are routinely used to follow the severity and progression of neuromuscular involvement and correlate with survival time, have not been investigated^33^,^34^.

Recently, our group reported the first clinico-pathologic evidence of retinal involvement in a patient with familial ALS due to a *C9orf72* mutation^35^. Peri-nuclear inclusions immunoreactive to anti-p62 antibody were found in the INL, reminiscent of those found in the hippocampus and cerebellum in this form of ALS, and were associated with decreased contrast sensitivity in this patient^36^.

This pathologic finding and the lack of consensus in OCT literature were the main stimulus for the current study. We hypothesized that a clinically similar ALS cohort will have macular changes detectable on OCT compared to age-matched controls and that retinal sub-layer thinning will correlate with measures of disease severity.

## Methods

### Ethics

This study was approved by the Northwestern University Institutional Review Board and all patients gave informed written consent. The study adhered to the tenets of the Declaration of Helsinki and all work was HIPAA-compliant.

### Subjects

Twenty-one patients who met the El Escorial criteria for definite ALS were recruited at Northwestern University^37^. Exclusion criteria included a diagnosis of a neurological disease other than ALS and ophthalmologic disease other than corrected refractive error, including glaucoma, confirmed by history and chart review. Patients were considered to have familial ALS if one or more of their first-degree relatives had a diagnosis of ALS. Imaging and clinical data from age-matched healthy subjects were provided by the Kellogg Eye Institute, University of Michigan.

Clinical data was collected retrospectively from the electronic medical record. Date of initial ALS symptom onset and anatomical site of disease onset were obtained from the first neurology clinic visit note. The forced vital capacity % predicted (FVC%), defined as FVC divided by the average FVC in a population of individuals with similar age, sex and body composition, upright forced expiratory volume in 1 second % predicted (FEV_1_%), defined as FEV1 divided by the average FEV1 in a population of individuals with similar age, sex and body composition, and ALS Functional Rating Scale (ALS-FRS-R) were obtained from the neurology clinic visit note with date closest to that of OCT acquisition. Eleven patients had OCT and clinical data collected in the same week. Mean and standard deviation of time between OCT and clinical data collection was 0.33 ± 32.3 days (positive value represents OCT occurring after clinical data collection; range = 77 days before to 53 days after neurology clinical data collection).

### Imaging and sub-layer segmentation

Macular scans of the right eye of each subject were obtained using the Spectralis spectral-domain OCT imaging platform (Heidelberg Engineering, Heidelberg, Germany). Macular scans were completed with horizontal scan lines covering a 6 × 6 mm area centered on the fovea. Eye tracking software was used to minimize motion artifact. Macular scans of age-matched control subjects performed on an identical OCT platform were provided by the Kellogg Eye Institute, University of Michigan.

Average macular sub-layer thickness within the entire 6 mm diameter Early Treatment Diabetic Retinopathy Study grid was measured semi-automatically, in two steps. First, an automatic layer segmentation software, Duke Optical Coherence Tomography Retinal Analysis Program (DOCTRAP), which accounts for various potential imaging artifacts and has been validated in multiple clinical trials, was used to delineate seven retinal layer boundaries on each horizontal B-scan^38^,^39^,^40^,^41^. These retinal layer boundaries defined 6 retinal sub-layers; macular RNFL, ganglion cell layer/inner plexiform layer (GCL/IPL), INL, outer plexiform layer/outer nuclear layer (OPL/ONL), inner segment/outer segment (IS/OS), and retinal pigment epithelium (RPE). This automated grading was followed by a quality control procedure to further validate these boundaries in the second step. All segmented layer boundaries were reviewed by a masked expert manual grader who, if needed, adjusted the boundary lines utilizing DOCTRAP’s graphical user interface. Finally, in order to correct any residual errors in segmentation, a second expert reviewed all the adjustments made by the first grader. Sub-layer thicknesses were automatically measured in each scan and average macular sub-layer thicknesses were calculated.

To assess the repeatability of sub-layer segmentation and thickness measurements, macular OCT scans from 4 ALS and 4 healthy control subjects were selected at random and underwent repeat measurement of the macular RNFL and GCL/IPL layers by the same reader. Initial and repeat measurements were compared and a concordance correlation coefficient was calculated.

### Statistical analysis

Statistical analyses were performed using the Statistical Package for the Social Sciences version 22 (SPSS Inc, Chicago, IL). Demographic data and OCT measurements were analyzed using chi-squared calculations or independent t-tests. Partial correlation coefficients controlling for age, gender, and when appropriate, days between OCT imaging and clinical data collection, were calculated to assess the correlations between FVC%, FEV_1_%, months since symptom onset, and the OCT measurements. A *P* value less than 0.05 was considered to be statistically significant.

## Results

### Patient Demographics

We enrolled 21 patients who had a diagnosis of ALS made by a neurologist with expertise in neuromuscular medicine. Four subjects had familial ALS while 17 had sporadic ALS. Of the 4 subjects with familial ALS, 2 carried mutations in the *C9orf72* gene and 2 carried mutation in the *CHCHD10* gene. Demographics and clinical factors of the ALS patients and healthy controls are displayed in Table 1.

### Optical Coherence Tomography Measurements

Macular OCT measurements of ALS and control cohorts are reported in Table 2. Shapiro-Wilk test demonstrated normal distribution (P > 0.05) of macular thickness (both ALS and control cohorts), FVC%, FEV_1_%, ALS disease duration, and ALS-FRS-R data. Total macular thickness was not significantly reduced in the ALS cohort compared to age-matched controls (302.2 ± 9.0 μm vs. 308.0 ± 14.1 μm *P* = 0.118). Sub-layer segmentation revealed significant thinning in the macular RNFL of the ALS cohort compared to controls (36.1 ± 3.5 μm vs 38.6 ± 3.7 μm, *P* = 0.029). No significant differences in GCL/IPL, INL, OPL/ONL, IS/OS or RPE layer thicknesses were found.

The concordance correlation coefficient comparing initial and repeat OCT sub-layer measurements in 4 ALS and 4 healthy control subjects was 0.9998.

### Correlation with Clinical Metrics

**Figure 1:**
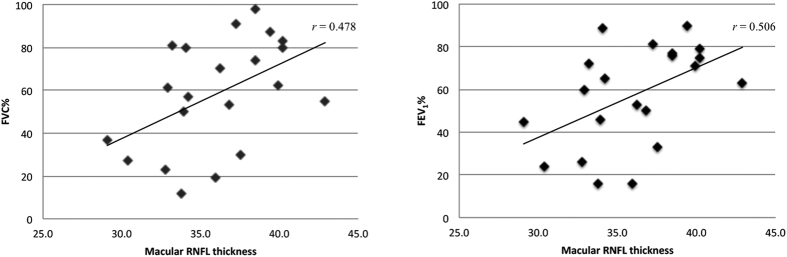
Scatter plot representing the correlation between Macular RNFL thickness and (A) Forced vital capacity percent predicted and (B) Forced expiratory volume in 1 second percent predicted. r value represents partial correlation coefficient controlling for age, gender, and time interval between OCT and clinical data collection.

Macular RNFL thickness in ALS patients correlated with FVC% and FEV_1_% while controlling for age and gender (r = 0.478, *P* = 0.045; r = 0.506, *P* = 0.032, respectively) (Table 3, Fig. 1). Total macular thickness did not correlate with FEV_1_% or FVC%. ALS-FRS-R correlated strongly with FVC% and FEV_1_% (r = 0.864, *P* = 0.001; r = 0.814, *P* = 0.001, respectively). Neither macular RNFL nor total macular thickness significantly correlated with disease duration or ALS-FRS-R.

## Discussion

The number of neuronal subtypes and neurological functions recognized as affected by ALS pathology is growing^1^,^2^,^3^,^4^,^5^,^6^. Here we present structural OCT data that is supportive of subtle anterior visual pathway involvement in ALS. We propose that these structural alterations highlight underlying retinal neuronal degeneration, specifically in ganglion cell axons, that may correlate with the visual function deficits observed in a previous ALS cohort^14^. Although the total difference in macular RNFL thickness in the ALS cohort was small (2.5 μm), the statistical power inherent to highly averaged OCT images comprising of tens of thousands of A-scans, coupled with the excellent reproducibility of sub-layer segmentation and repeatability of the measurements, and the level of statistical significance found here are all supportive of the validity of the findings in this cohort. Reports of other retinal degenerative disorders have similarly identified subtle but statistically significant sub-layer thinning using OCT^42^,^43^. Furthermore, we identify a significant, positive correlation between macular RNFL thickness and FVC%, a clinical test that assesses neuromuscular involvement at the diaphragm and is predictive of mortality. No correlations between OCT measurements and disease duration or ALS-FRS-R were identified. It is unclear why macular RNFL correlated with pulmonary function tests but not disease duration or functional rating score. One potential explanation may be that the first two represent a more direct, quantitative measure of neurodegeneration (loss of ganglion cell axons and motor innervation of the diaphragm, respectively) while the relationship between neuronal cell loss and disease duration or functional ability may be more variable and dependent on an individual’s disease course.

The RNFL sub-layer thinning we observed is similar to that recently reported in the studies by Ringelstein *et al*. and Hubers *et al*., however we did not find significant changes in total macular or INL thicknesses^30^,^32^. Furthermore, we examined macular RNFL while Ringelstein *et al*. measured peripapillary RNFL. Important differences exist between the respective cohorts, including percent of patients with El Escorial definite ALS (100% current study vs 83% [Ringelstein *et al*.] vs unreported [Hubers *et al*.]). Additionally, our ALS cohort included familial ALS patients and had a longer average disease duration (44.4 ± 42.7 vs 22.3 ± 13.0 [Ringelstein *et al*.] vs 12 [Hubers *et al*.] months). Finally, other complex factors including genetic and environmental disease triggers make comparing heterogeneous ALS cohorts difficult. These cohort differences may partially explain the alternative conclusions regarding total macular and INL thinning between studies; RNFL involvement appears to be the most consistent finding. Given the small scale of the thickness changes seen here and in the other OCT studies in ALS, studies with larger cohort sizes or meta-analyses including studies with robust segmentation error correction methods will be necessary to further confirm the OCT sub-layer thinning patterns.

One of the current challenges of studying ALS patients is the lack of quantitative tools to measure disease severity and progression. Multiple methods have been investigated including metabolic, growth factor and inflammatory biomarker in plasma and CSF, as well as diffusion MR imaging of the CNS^44^,^45^,^46^. OCT offers the advantage of directly imaging CNS neurons in a relatively cost-effective and non-invasive way. Correlation of RNFL thickness with pulmonary measures of ALS disease severity suggests OCT may be useful in following ALS progression. We have recently shown pathologic peri-nuclear inclusions in the INL of a patient with fALS and C9ORF72 mutation^35^. Our current study further supports ALS retinal involvement and suggests that RNFL thinning may provide a quantitative biomarker of ALS-related neurodegeneration. Following further confirmatory studies, a multi-pronged approach including clinical functional scales, plasma and CSF biomarkers, and direct imaging with OCT may serve as a more robust method of grading ALS severity.

Our findings of specific sub-layer thinning may represent a unique susceptibility of the RNFL to ALS pathology. Interestingly, recent findings have demonstrated that long axons have a higher susceptibility to ALS related neurodegeneration, which offers one hypothesis for why ganglion cell axons may be disproportionally affected in the retina^47^. A second potential link between ALS and the RNFL exists as mutations in optineurin, a gene involved in autophagy regulation, have been linked to both primary open-angle glaucoma and ALS^48^,^49^,^50^. Further investigation of genetic and mechanistic similarities, specifically in autophagy disregulation, may identify further overlap between the two neurodegenerative disorders.

Limitations of this study include the small sample size and cross sectional nature. An additional limitation is that OCT images and neurologic clinical data were not always obtained on the same day, however over half of the cohort had OCT imaging and neurology clinic visits within a span of 3 days and the entire cohort had a median of 0 days interval between these 2 data points.

Future longitudinal studies are needed to more completely evaluate the relationship between structural changes on OCT and ALS severity, patient function and disease progression. Analysis of regional thickness results, including ETDRS sectors, may also be pursued to identify any localized thickness changes likely not detected in the averaged approach used in this study. Additionally, ALS subgroup comparisons, including bulbar vs spinal onset, familial vs sporadic inheritance, and juvenile vs adult onset, with an appropriately powered cohort study or meta-analysis may help determine if sub-layer thinning is limited to certain ALS disease subtypes. Visual function tests such as contrast sensitivity, which has been shown to correlate with RNFL thinning in MS, may prove capable of detecting retinal dysfunction that precedes structural changes and should be considered in future studies^51^. Such tests may also be useful, along side OCT imaging and standard neurologic examination, for disease screening and severity stratification.

## Additional Information

**How to cite this article**: Simonett, J. M. *et al*. Macular sub-layer thinning and association with pulmonary function tests in Amyotrophic Lateral Sclerosis. *Sci. Rep.* **6**, 29187; doi: 10.1038/srep29187 (2016).

## Figures and Tables

**Table 1:** Demographics and clinical factors for adult-onset ALS patients and healthy controls.

Characteristics	ALS (*n* = 21)	Healthy Controls (*n* = 21)	*P*
Age	55.2 ± 10.5	56.5 ± 12.0	0.716
Male sex	15 (71.4%)	9 (42.9%)	0.061
Months since symptom onset	43.2 ± 43.4 (range = 10–197)	NA	
Predicted upright FVC%	58.6 ± 25.7	NA	
Predicted upright FEV-1%	57.5 ± 23.6	NA	
ALS-FRS-R	28.1 ± 12.5	NA	
Disease onset location			
Bulbar	3 (14.3%)	NA	
Spinal–right sided	11 (52.4%)	NA	
Spinal–left sided	5 (23.8%)	NA	
Spinal–bilateral	2 (9.5%)	NA	

FVC%, forced vital capacity percent predicted; FEV_1_%, forced expiratory volume in 1 second percent predicted. ALS-FRS-R, ALS Functional Rating Scale.

**Table 2:** Total macular and sub-layer thicknesses of adult-onset ALS patients and Healthy controls.

	ALS patients (21)	Healthy Controls (21)	*P*
RNFL	36.1 ± 3.5	38.6 ± 3.7	**0.029**
GCL/IPL	70.0 ± 4.9	71.2 ± 5.6	0.467
INL	31.9 ± 1.9	31.7 ± 2.7	0.783
OPL/ONL	103.0 ± 5.9	104.6 ± 7.5	0.447
IS/OS	33.8 ± 1.6	32.7 ± 2.4	0.090
RPE	27.3 ± 3.0	29.1 ± 3.6	0.085
Total	302.2 ± 9.0	308.0 ± 14.1	0.118

All measurements reported in microns. RNFL, macular retinal nerve fiber layer; GCL/IPL ganglion cell layer/inner plexiform layer; INL, inner nuclear layer; OPL/ONL, outer plexiform layer/outer nuclear layer; IS/OS, inner segment/outer segment; RPE, retinal pigment epithelium. Bolded *P* values are statistically significant (*P* < 0.05).

**Table 3:** Partial correlation between OCT measurements and adult-onset ALS disease duration while controlling for age, gender, and time interval between OCT and clinical data collection.

	Total macula		RNFL	
FVC%	r = 0.204	*P* = 0.428	r = 0.478	*P* = **0.045**
FEV_1_%	r = 0.231	*P* = 0.357	r = 0.506	*P* = **0.032**

RNFL, macular retinal nerve fiber layer; FVC%, forced vital capacity percent predicted; FEV_1_%, forced expiratory volume in 1 second percent predicted; r, partial correlation coefficient. Bolded *P* values are statistically significant (*P* < 0.05).
